# A scoping review of outcome measures for people living with dementia and family supporters to evaluate Recovery College dementia courses

**DOI:** 10.3389/fpsyt.2025.1591772

**Published:** 2025-05-06

**Authors:** Jarin Alam, Juniper West, Esme Moniz-Cook, Emma Wolverson, Melanie Handley, Linda Birt, Chris Fox

**Affiliations:** ^1^ Research and Development, Norfolk and Suffolk NHS Foundation Trust, Norwich, United Kingdom; ^2^ Faculty of Health Science, University of Hull, Hull, United Kingdom; ^3^ The Geller Institute of Ageing and Memory, The University of West London, London, United Kingdom; ^4^ Centre for Research in Public Health and Community Care, University of Hertfordshire, Hatfield, United Kingdom; ^5^ School of Healthcare, University of Leicester, Leicester, United Kingdom; ^6^ Medical School, University of Exeter, Exeter, United Kingdom

**Keywords:** dementia, outcome measure, personal recovery, recovery college, positive psychology, hope, resilience, empowerment

## Abstract

**Introduction:**

Recovery Colleges (RC/RCs) aim to promote personal recovery through co-produced courses, grounded in the CHIME (Connectedness, Hope, Identity, Meaning, Empowerment) framework. The DiSCOVERY research programme noted that RC dementia courses may offer a person-centred approach to post-diagnostic dementia care. However, the lack of validated outcome measures for this context presents a challenge in evaluating RCs’ effectiveness. This scoping review examines the potential outcome measures for evaluating the impact of RC dementia courses.

**Methods:**

The review followed the Arksey and O’Malley framework, searching for eligible papers across six databases related to dementia and the CHIME strengths-based approach. Instruments relating to personal recovery and positive psychology for people with dementia or their family supporters were included. Measures of cognition, clinical symptoms, or ‘negative constructs’ (e.g., burden) were excluded. DiSCOVERY stakeholder groups (people with dementia and clinicians) met to collaboratively identify meaningful domains and relevant measures.

**Results:**

Fourteen instruments relating to hope, resilience, self-efficacy, empowerment, and coping were identified. Stakeholders of people living with dementia endorsed domains of empowerment, resilience, and hope. No single instrument captured the range of outcomes that underlie the concepts of the RC dementia course.

**Discussion:**

This study contributes to the limited literature on instruments for the evaluation of concepts underlying RC dementia courses. Findings suggest a need for adaptation and further validation of existing measures, to address responsiveness, interpretability, and the inclusion of domains related to recovery. Future research on recovery in the context of dementia should involve developing or adapting new measures, conducting feasibility studies, and exploring cultural sensitivity for diverse populations.

## Introduction

Recovery Colleges (RC/RCs) are delivered in the UK by the National Health Services (NHS). They offer courses designed to support personal recovery and self-management amongst individuals experiencing mental health challenges. These courses are co-produced by people with lived experience and mental health professionals, providing opportunities for collaboration and skill sharing, aimed at enhancing healthcare outcomes beyond symptom reduction (1). RCs are inclusive of family supporters, healthcare staff, and people with lived experience (2). Course attendees are referred to as ‘students’ and facilitators as ‘tutors.’ This is thought to empower course attendees by fostering a community-based model that promotes empowerment, learning, and growth. The structure aims to create equitable relationships between people with ‘lived’ (i.e., people with a ‘diagnosis’/family supporters) and ‘learned’ (i.e., professionals working in services) experiences of mental health, potentially breaking down stigma-related barriers of ‘them’ and ‘us’ (1, 3) or related power imbalances.

The overall aim of RCs is to empower individuals to rebuild meaningful lives alongside their diagnoses, by focussing on strengths and promoting hope, identity, and connection (4). The conceptual framework CHIME (i.e., Connecting with others, inspiring Hope, maintaining a positive Identity, finding Meaning in life outside of symptoms, and Empowering control over life) underpins a strengths-based approach (5). In line with these principles, assessment tools used in this context should reflect positive psychological and recovery-oriented outcomes—such as wellbeing, purpose, and self-efficacy—rather than focussing only on decline or impairment. People living with a health condition, and their unpaid supporters, provide unique and inspiring perspectives derived from their lived experience when participating in RC courses as ‘peer tutors’ or ‘experts by experience,’ sharing decision-making during co-production and co-facilitation (4, 6).

Building on established UK NHS infrastructure, RC dementia courses offer a novel approach to post-diagnostic dementia support, where co-production is central to delivery (7) thus promoting a focus on thriving with dementia that promotes an optimistic and empowering outlook, following a diagnosis. The DiSCOVERY research programme (NIHR131676,2022–2024) aimed to understand what works post-diagnosis, for whom, in what circumstances, and why, in RC dementia courses (8). Findings of a realist review, which aimed to develop an initial programme theory, supported the application of personal recovery principles for people with dementia, their unpaid supporters, and staff, emphasising the importance of diverse stakeholder inclusivity during co-production and co-facilitation (9). A national survey of RC dementia courses which located 12 NHS-funded courses across the UK (10) found barriers in delivery such as limited organisational awareness of RCs, low ethnic diversity in attendees, and confusion about the term ‘recovery’ in the context of a progressive and life-limiting illness. Nonetheless, staff endorsed the value of co-produced dementia courses and suggested strategies to overcome these barriers (10).

Mental health outcome measures, reported by patients, clinicians, or sometimes by a proxy like a family member, are often used to assess an individual’s status in evaluating interventions. For example, outcome domains may include social (11) inclusion (12, 13), mood or behavioural symptoms (14), wellbeing (15), and staff confidence in working with people with dementia (16). If we are to measure effectiveness, outcome measures need to be matched to the concept being evaluated (17); in this case, the CHIME framework may be a useful guide to identifying outcomes. Self-report outcome measures are also appropriate in this context, aligning with recovery-oriented practices and principles of RC, relating to empowering and engaging individuals in their personal recovery process (5, 18). Positive psychology, which scientifically studies what makes life most worth living by focussing on strengths, virtues, and factors that contribute to human flourishing and wellbeing, closely aligns with CHIME. CHIME emphasises personal strengths and resilience to enhance wellbeing (17). For example, the focus on fostering hope and positivity and finding meaning in the CHIME framework reflects the concepts of hope and optimism for the future and building meaningful lives within positive psychology (19, 20). Given these overlaps, identifying positive psychology outcome measures for use in RC dementia courses may be a useful way to measure aspects of personal recovery. Other reviews have reported on existing self-report positive psychology measures used (17), validated or adapted for use by people with dementia or family supporters (21–23), but little is known about how these relate to the concept of CHIME that underlies RCs in the context of dementia. Co-researching with key patient and public involvement groups, including service users with dementia, ensures research that is being co-produced is relevant and meaningful for people with dementia (24, 25). Significantly, their lived expertise can strengthen the validity of the results and accessibility of the outputs. Co-creating RC evaluative work is also emerging, but few peer-reviewed articles can be found (26).

In dementia research, several core outcome sets have been developed with and for people living with dementia, which identify outcomes that are highly valued by them (27). This scoping review aims to build on this work by identifying outcome measures for people living with dementia and their family supporters as either attendees or peer tutors of RC dementia courses. This may help service providers to evaluate the impact of this type of post-diagnostic service.

The objectives were to

1. Identify domains of outcomes for people with dementia and their supporters (including friends) attending or co-facilitating RC dementia courses, guided by the realist programme theory.
2. Review literature to identify standardised outcome measures that align with these outcome domains.
3. Evaluate the measurement properties of the instruments.
4. Present stakeholders (people with dementia and clinicians) with an overview of domains and related instruments to discuss, refine, and co-produce recommendations for accessible outcome measures for evaluating RC dementia courses.

## Methods

### Study design

The scoping review methodology was employed as such reviews are useful in areas with emerging or fragmented evidence, as they provide a comprehensive overview of existing research and highlight gaps in knowledge (28). The review adhered to the PRISMA-ScR checklist (29) to ensure rigorous and transparent reporting of the review process. We followed the six-step framework proposed by Arksey and O’Malley (30) with an adapted first step.

#### Step 1: embedded stakeholder engagement

The Arksey and O’Malley (30) framework originally described ‘consultation with relevant stakeholders’ as an optional last stage. The other authors’ scoping RCs and co-creation modified the first step reconceptualising this as ‘co-creator’ engagement (26). Our study also involved co-researching throughout—renamed ‘embedded stakeholder engagement’—to reflect integrated processes throughout the entire co-produced DiSCOVERY research programme. The initial domains for the scoping review were drawn from the realist programme theory of dementia courses involving co-researching with DiSCOVERY stakeholders of people with lived experience of dementia (the Partners in Research group) which consisted of up to 12 members of people either living with dementia and family supporters and healthcare professionals (the Staff Advisory group) to validate the findings (8, 9).

For stakeholder engagement within the scoping review, the research team first grouped outcome measures into higher-level domains to guide discussions. Then, the team collaborated with DiSCOVERY stakeholders to identify the most relevant domains that would be meaningful for RC dementia courses. This ensured that the lived experiences of people with dementia and their supporters were not overlooked and occurred in two group discussions where higher-level domains of the eligible outcome measures extracted from the literature search were presented and participants considered what domains might be meaningful for them. The domains were broadly related to knowledge of dementia, coping, hope, stigma, resilience, empowerment, self-efficacy, social support, and wellbeing. These discussions aided the ranking of the importance of each domain for both people with dementia and supporters, as each person was asked to select their top 3 domains by importance, in which the average ranking was calculated to identify the most relevant outcome measures for dementia courses.

#### Step 2: identifying the review question

The following review questions were developed:

- What existing outcome measures, if any, can be used to evaluate the experiences of people living with dementia either attending or co-facilitating Recovery College dementia courses?
- What existing outcome measures, if any, can be used to evaluate the experiences of family/friend supporters of people living with dementia either attending or co-facilitating Recovery College dementia courses?

#### Step 3: identifying relevant studies

##### Search strategy

The review terms were guided by the outcome domains identified in the RC realist programme theory (9) and discussions with stakeholder groups which included ‘lived’ and ‘learned’ experts. To manage the two distinct perspectives, two separate searches were conducted: one for people living with dementia and another for family supporters. Members of the DiSCOVERY team (JA, LB, and JW) met to ensure the search terms were consistent with the results of the realist review. Domains included ‘personal recovery’ constructs based on the CHIME recovery framework (5) and positive psychology constructs.

Comprehensive search strategies were designed with input from the DiSCOVERY project team, including a librarian (RK). Between August 2024 and November 2024, the following academic databases were searched to identify relevant studies by JA: Cochrane Library, MEDLINE, PubMed, PsycINFO, CINAHL, and Embase. To capture a broad spectrum of relevant literature across various disciplines, an expansive and inclusive search strategy was used (31).

##### Search terms

To ensure the inclusion of all available and relevant preliminary studies, this study used Medical Subject Headings (MeSH) terms to identify studies concerning the measured constructs. The following key terms were applied to each database for both people living with dementia and family supporters: dement* or Alzheimer*. Search terms relevant to outcome measures and psychometric studies were also included: outcome* OR measur* OR evaluation OR assessment* OR questionnaire* OR patient-report* OR tool* OR index OR self-report OR scale OR inventor* OR instrument AND validation OR develop* OR psychometric.

Additional search terms of outcomes for people with dementia included the following: person-cent* OR stigma OR self-stigma OR motivat* OR belong* OR flourish* OR positive psychol* OR optimis* OR connect* OR social engage* OR “social relationship” OR recover* OR accept* OR agency OR control OR empower* OR self-esteem OR meaning OR purpose OR identity OR strength* OR resilien* OR self-efficacy OR hope* OR education OR knowledge OR peer OR autonomy OR “positive affect” OR self-agency OR self-acceptance OR self.

To see the additional search terms used for family supporter outcomes and an example of the full search strategy, see **Supplementary File A**. For an example of the full search strategy used for people with dementia, see **Supplementary File B**. Search terms were limited to titles and abstracts to allow screening of a large number of articles and identify those most likely to address the research question.

#### Step 4: selection of studies

##### Screening

**Figure 1:**
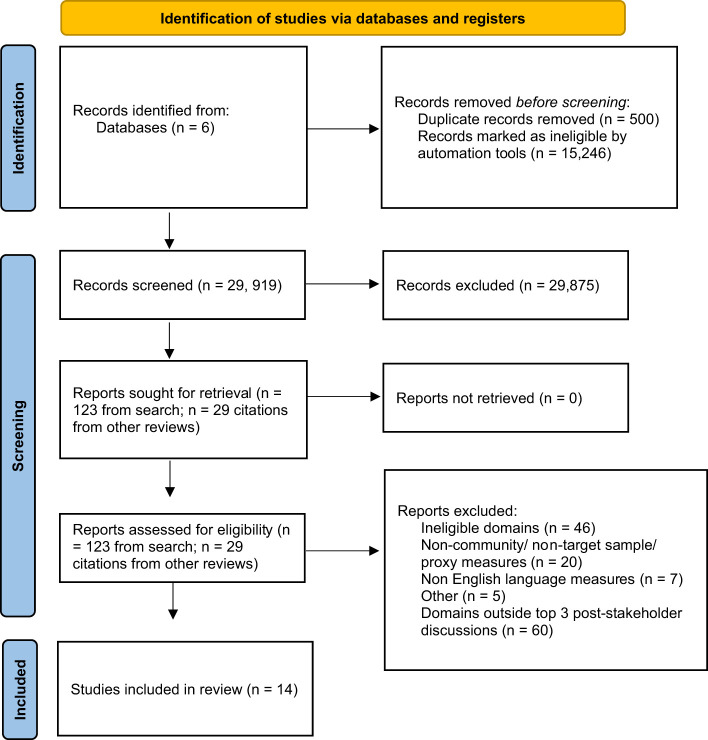
summarises each stage of the screening process.

After downloading the citations into RefWorks, JA independently reviewed all titles and abstracts to determine eligibility based on the criteria outlined below (**Figure 1**). These criteria were identified through discussion and refinement with the research team. Reference lists of key articles and reviews were manually searched to identify relevant research missing from database searches (17, 21–23, 32–35). JA independently reviewed all eligible full-text articles. A second reviewer (EW) assessed a 20% random sample of the full-text articles for consistency in the application of the criteria, ensuring the reliability and validity of the review. Any discrepancies during the screening process were discussed amongst the research team to determine the inclusion or exclusion of the literature in question.

##### Eligibility criteria

The following inclusion criteria were applied: 1) sample: participants must have a diagnosis of dementia or be a family member or friend supporting a person with dementia; 2) target user of outcome measure: the study must explicitly state that the outcome measure is either to be completed by people with dementia or their supporters; 3) study design: primary research studies within peer-reviewed literature describing the development and/or validation of outcome measures for research with the target population; 4) setting: validated for use in community-based settings; 5) types of outcome measures: self-report questionnaire-based outcome measures within domains of positive psychology or personal recovery; 6) year: studies conducted between 1998 and 2024; and 7) language: papers published in English.

The exclusion criteria included the following: 1) studies that focussed on clinical outcomes such as cognitive function or behaviour and psychological symptoms of dementia; 2) outcome domains measuring deficits/negative constructs like caregiver burden or generic outcome measures of quality of life; 3) studies focussing on conditions other than dementia; 4) intervention studies unless it explores development or validation process; and 5) studies using proxy-reported outcome measures. After screening the literature, an additional restriction was added to exclude papers that did not use English-language versions of the outcome measures.

These criteria were selected to ensure that the outcome measures included were relevant and appropriate for people with dementia and their supporters and aligned with the ethos of Recovery Colleges. Prioritising self-report tools, validated in community settings and grounded in positive psychology or personal recovery, ensured a focus on strengths-based, person-centred outcomes rather than clinical or deficit-based constructs. Limiting studies to those reporting development or validation processes helped streamline the review by narrowing the scope to outcome measures with established psychometric properties, enhancing both the rigour and efficiency of the literature selection. The timeframe corresponds with the reintroduction of positive psychology (36), and English-language restrictions were applied for feasibility. Exclusions further ensured conceptual alignment by removing studies that addressed unrelated populations, proxy measures, or outcomes not reflective of the CHIME framework or positive psychology.

#### Stage 5: charting the data

##### Data extraction

JA extracted data using a pre-determined Excel spreadsheet template extracting the authors, study design, study aim, target user, age and sample size, and geographical location. Other information relevant to the outcome measures was collected, including the name of the measure, domains assessed, number of items, and response options. Information on psychometric properties (e.g., content validity, internal consistency, construct validity, reliability, responsiveness) was also extracted. Measures were grouped into domains relevant to the CHIME framework and positive psychology constructs.

##### Quality appraisal

As the review focussed on development and validation studies, we ordered the ratings of instruments using Terwee’s criteria and its tool for quality assessment, covering content validity, internal consistency, construct validity, reproducibility, responsiveness, floor and ceiling effects, and interpretability (37; **Table 1**). This tool has been used in other similar studies (e.g., 21). Each outcome measure is rated on a scale (0 to 18), with scores categorised as poor (0–4), moderate (5–9), good (10–14), or very good (15–18) (22). JA appraised the risk of bias in all eligible studies and LA independently assessed 25%. Discrepancies in ratings were discussed and resolved with a third researcher (EW). We viewed useful measures for evaluating RC courses as those with the highest quality ratings.

**Table 1:** Terwee’s criteria (2007).

Property	Definition	Quality criteria
1. Content validity	The extent to which the domain of interest is comprehensively sampled by the items in the questionnaire (the extent to which the measure represents all facets of the construct under question).	+ 2 = A clear description of measurement aim, target population, concept(s) being measured, and item selection AND target population (investigators OR experts) were involved in item selection. ? 1 = A clear description of the above aspects is lacking OR only the target population was involved OR doubtful. − 0 = No target population involvement. 0 0 = No information found on target population involvement.
2. Internal consistency	The extent to which items in a (sub)scale are inter-correlated, thus measuring the same construct.	+ 2 = Factor analyses performed on an adequate sample size (7× number of items, ≥100) AND Cronbach’s alpha(s) calculated per dimension AND Cronbach’s alpha(s) between 0.70 and 0.95. ? 1 = No factor analysis OR doubtful design or method. − 0 = Cronbach’s alpha(s) <0.70 or >0.95, despite adequate design and method. 0 0 = No information found on internal consistency.
3. Criterion validity	The extent to which scores on a particular questionnaire relate to a gold standard.	+ 2 = Convincing arguments that the gold standard is ‘gold’ AND correlation with the gold standard ≥0.70. ? 1 = No convincing arguments that the gold standard is ‘gold’ OR doubtful design or method. − 0 = Correlation with gold standard <0.70, despite adequate design and method. 0 0 = No information found on criterion validity.
4. Construct validity	The extent to which scores on a particular questionnaire relate to other measures in a manner that is consistent with theoretically derived hypotheses concerning the concepts being measured.	+ 2 = Specific hypotheses were formulated AND at least 75% of the results are in accordance with these hypotheses. ? 1 = Doubtful design or method (e.g., no hypotheses). − 0 = Less than 75% of hypotheses confirmed, despite adequate design and methods. 0 0 = No information found on construct validity.
5. Reproducibility		
5.1 Agreement	The extent to which scores on repeated measures are close to each other (absolute measurement error).	+ 2 = SDC < MIC OR MIC outside the LOA OR convincing arguments that agreement is acceptable. ? 1 = Doubtful design or method OR (MIC not defined AND no convincing arguments that agreement is acceptable). − 0 = MIC ≤ SDC OR MIC equals or inside LOA despite adequate design and method. 0 0 = No information found on agreement.
5.2 Reliability	The extent to which patients can be distinguished from each other, despite measurement errors (relative measurement error).	+ 2 = ICC or weighted Kappa ≥0.70. ? 1 = Doubtful design or method. − 0 = ICC or weighted Kappa <0.70, despite adequate design and method. 0 0 = No information found on reliability.
6. Responsiveness	The ability of a questionnaire to detect clinically important changes over time.	+ 2 = SDC or SDC < MIC OR MIC outside the LOA OR RR >1.96 OR AUC ≥0.70. ? 1 = Doubtful design or method. − 0 = SDC or SDC ≥ MIC OR MIC equals or inside LOA OR RR ≤1.96 OR AUC <0.70, despite adequate design and methods. 0 0 = No information found on responsiveness.
7. Floor and ceiling effects	The number of respondents who achieved the lowest or highest possible score.	+ 2 = ≤15% of respondents achieved the highest or lowest possible scores. ? 1 = Doubtful design or method. − 0 = >15% of the respondents achieved the highest or lowest possible scores, despite adequate design and methods. 0 0 = No information found on interpretation.
8. Interpretability	The degree to which one can assign qualitative meaning to quantitative scores.	+ 2 = Mean and SD scores presented for at least four relevant subgroups of patients AND MIC defined. ? 1 = Doubtful design or method OR fewer than four subgroups OR no MIC defined. 0 0 = No information found on interpretation.

a + = positive rating; ? = indeterminate rating; − = negative rating; 0 = no information available. b Doubtful design or method = lacking a clear description of the design or methods of the study, sample size smaller than 50 subjects (should be at least 50 in every (subgroup) analysis), or any important methodological weakness in the design or execution of the study.

MIC, minimal important change; SDC, smallest detectable change; LOA, limits of agreement; ICC, intraclass correlation; SD, standard deviation.

##### Ethical approval

This scoping review of published literature did not involve primary data collection from research participants. However, the scoping review methodology draws on deliberation and shared decision-making with key stakeholders. The review was completed with the Partners in Research group and the Staff Advisory group, who have been involved in every stage of the study. Informed consent has been obtained for their involvement (ethics reference numbers DiSCOVERY WP1 22/WM/0215 and DiSCOVERY WP2-4 22/WM/0021).

## Results

### Discovery stakeholder discussions

Discovery stakeholders, including those with lived and learned experience of dementia, collaboratively identified suitable domains and outcome measures for evaluating RC dementia course outcomes. This ensured that measures captured relevant domains for attendees or co-facilitators and highlighted areas for development. The inclusion of CHIME and positive psychology domains was shaped by discussions with DiSCOVERY stakeholders, who emphasised the importance of measuring personal recovery for both people with dementia and their family supporters. Hope was recognised as an important outcome for both groups, along with a strong focus on resilience by people with dementia. Empowerment, specifically the ability to regain control over one’s life and maintain independence after the dementia diagnosis, was also identified as a critical outcome for people with dementia. In addition to hope, the importance of increasing coping strategies and increasing confidence in managing the challenges of supporting someone living with dementia throughout their journey was identified as essential for caregivers.

Our review of instruments and stakeholder advice led us to concentrate on outcome measures relevant to the three top ranking domains which were also aligned with some aspects of the CHIME framework (see **Supplementary File C**). Rankings of outcome domains were also based on discussions with stakeholders, including clinicians and people with dementia to reflect their collective perspectives on the importance of each domain for evaluating RC dementia courses. Therefore, these rankings were grounded in lived experience and expert input. Measures assessing social support, stigma, and dementia knowledge were therefore excluded.

### Overview of outcome measures

The top 3 domains for each population, people with dementia and family supporters, are reported here as they fall within the domains of the CHIME framework and/or positive psychology. A total of 14 outcome measures relevant to evaluating Recovery College dementia courses were identified, focussing on the domains of hope, resilience, self-efficacy, empowerment, and coping. **Table 2** summarises the 14 outcome measures, providing key study information and their psychometric properties.

**Table 2:** Overview of outcome measures.

Domain: Coping						
Author (Year), Country	Measure	Participants; Age	Number of items	Response option	Reliability	Validity
Cooper et al. (38), UK	Brief COPE	Carers of people with dementia (n=125); Mean age = 63.8 (SD = 13.3), range 30 – 90	14-item instrument (subscales: emotion-focussed; problem focussed; problem-focussed)	4-point Likert scale	Internal consistency: At Time 1, emotion-focused subscale α = 0.72, problem-focused subscale scale α = 0.84, dysfunctional coping scale α = 0.75. Test-retest: Overall sample: Time 1 correlated with Time 2 (r = 0.67) and Time 3 (r = 0.54), p < 0.001. Stable burden subgroup (burden score within 1 SD from Time 1 to Time 3): Time 1 correlated with Time 2 (r = 0.72) and Time 3 (r = 0.57), p < 0.001. Subscale reliability: Dysfunctional, Emotion-Focused, and Problem-Focused Coping: T1-T2 (overall sample): r = 0.64, 0.51, 0.71, p < 0.001. T1-T2 (stable burden group): r = 0.68, 0.58, 0.72, p < 0.001. T1-T3 (overall sample): r = 0.59, 0.44, 0.38, p < 0.001. T1-T3 (stable burden group): r = 0.59, 0.60, 0.46, p < 0.001.	Construct validity: Emotion-focused coping was positively associated with problem-focused coping (r = 0.68, p < .001). Dysfunctional coping was predicted by higher burden (β = 0.36, p < .001) and problem-focused coping (β = 0.31, p = .003). Problem-focused coping was positively associated with avoidant attachment (r = 0.40, p < .001), ADL impairment (r = 0.22, p < .01), and social support (β = 0.10, p = 0.25). Content validity: No target user involvement during development (39).
Domain: Empowerment						
Stoner et al. (23), UK	Control, autonomy, self-realisation and pleasure scale	People with dementia (n = 225); Mean age = 77.1 (SD = 9.4), range 50–99	19-item instrument	4-point Likert scale	Internal consistency: Overall α = .86; Control subscale α = .645, autonomy subscale α = .505, pleasure subscale α = .718, self-realisation subscale α = .781. Test-retest (one week; n = 48): ICC = 0.86 (95%CI = 0.76 - .92)	Convergent validity: Hope and resilience (r = 0.73, p < 0.01) Engagement and independence (r = 0.75, p < 0.01) Concurrent validity: Quality of life (r = 0.71, p < 0.01) Discriminant validity: Depression (r = –0.71, p < 0.01). Content validity: No target user involvement (39).
Stoner et al. (40), UK	Engagement and Independence in Dementia Questionnaire	People living with dementia (n = 225); Mean age = 77.1 (SD = 9.4), range 50-99	26-item instrument (Subscales: activities of daily living; decision-making; activity engagement; support; reciprocity)	5-point Likert scale	Internal consistency: Overall EID-Q α = .91, Sense of independence subscale α = .85, and social engagement subscale α = .85. Test-retest: ICC = 0.77, (95% CI = 0.61 - 0.87, p < .001)	Convergent validity: Quality of life with EID-Q (r =.68, p<.001), independence subscale (r = .63, p<.001), engagement subscale (r = .69, p<.001). CASP-19 with EID-Q (r = .75, p<.001), engagement subscale (r = .66, p<.001), independence subscale (r = .70, p<.001). Divergent validity: Depression with EID-Q (r = -.74, p<.001), independence subscale (r = -.70, p<.001), engagement subscale (r = -.74, p<.001). Factor analysis: CFA supported 5-factor second-order model: χ²(293) = 694, p < .001; CFI = 0.83; RMSEA = 0.08; SRMR = 0.07.
Menne et al. (41), USA	Decision-Making Involvement Scale	People with dementia (n=217); Mean age = 76 (SD = 9.2)	15-item instrument (15 dimensions of the person with dementia’s day-to-day decision making.)	People with dementia report on their decision-making involvement on a 4-point scale:	Internal consistency: α = 0.85 (person with dementia) Test-retest: Not reported	Factor Structure: 1-factor solution; explained variance = 33.74%. Convergent Validity: Quality of life (r = 0.30, p < .10). Divergent Validity: No significant correlation with depression scores or relationship strain. Content validity: Stakeholder involvement during development (42) Other: People with dementia reported moderate involvement in decision-making (M = 2.29, SD = 0.60). Note: proxy measure for caregivers has been excluded for this review.
Domain: Caregiver Self-efficacy						
Steffen et al. (43), USA	The Revised Scale of Caregiving Self-efficacy	Study 1: Female Supporters of people with dementia (n=169); Mean age = 63.8 (SD = 8.3) Study 2: Supporters of people with dementia (n=145); Study 2: Mean age = 60.2 (SD = 13.3)	15 items (subscales: obtaining respite; responding to disruptive patient behaviours; controlling upsetting thoughts about caregiving)	Likert scale 0 to 100; Higher scores reflect greater self-efficacy	Internal consistency: Obtaining respite subscale α = 0.85, Responding to behaviours subscale α = 0.82, controlling upset thoughts subscale α = .85. Test-retest α = 0.70–0.76 for the three subscales. Test-retest reliability in Study 2 (two-weeks later): r = .76 for Obtaining respite self-efficacy, r = .70 for self-efficacy subscale Disruptive patient behaviour self-efficacy, r = .76 Controlling Upsetting thoughts self-efficacy.	Factor analysis: a three-factor model fit, was found with a CFI of 0.93. Convergent validity: Perceived social support (r = 0.47) Divergent validity: Depression (r = 0.38), Anger (r = −0.45), and Anxiety (r = −0.37). Content validity: Some evidence of stakeholder involvement for administration format but not item content (44)
Kuhn and Fulton (45), USA	Self-efficacy Scale	Supporters of people with dementia (n = 45); Mean age = 74.3 (SD = 7.4), range 25 - 83	15-item instrument	5-point Likert scale	Internal consistency: α = 0.90	Convergent validity: Not reported Effectiveness: Significant increase in caregiver self-efficacy scores (Pre: M = 3.0, SD = 0.7; Post: M = 3.3, SD = 0.7) t(43) = -4.619, p < .0005. Effect size: d = 0.4 (moderate)
Vernooij‐Dassen et al. (46), The Netherlands	Short Sense of Competence Questionnaire	Supporters of people with dementia (n=141); Mean age = 63	7-item instrument	5-point Likert Scale.	Internal consistency: α = 0.76 Additional reliability testing in three other studies: α = 0.68 to 0.87	Construct validity: Sense of competence Questionnaire (SCQ; 47) (r = 0.88). Content validity - No involvement of target population.
Jansen et al (48), The Netherlands	Sense of competence questionnaire	Supporters of people with dementia symptoms (i.e. cognitive impairment, pre-diagnostic dementia or dementia in its early stages; n=93); Mean age = 62.9 (SD =14.4), range 32.5–91.2	27 item instrument (subscales: satisfaction with the care recipient; satisfaction with one’s own performance as a caregiver; consequences of involvement in care for the personal life of the caregiver)	5-point scale	Internal consistency: Satisfaction with care recipient α = 0.83, Satisfaction with own performance α = 0.83, Consequences of involvement in care: α = 0.85.	Construct validity: Most hypothesis rejected except for subscale consequences of involvement in care with burden (r = -0.69, 95% CI: 1.00 0.62) and mental QOL (r = 0.44, 95% CI:0.14, 0.57). Factor Structure: 3-factor model confirmed Ceiling Effects: 18% of participants reached the maximum score on the "Satisfaction with care recipient" subscale Overall SCQ Score: Mean = 107.7, SD = 13.7 Content validity: No involvement of unpaid caregivers during development (47)
Gottlieb and Rooney (49), Canada	RIS eldercare self-efficacy scale	Caregivers of people with dementia (n=146); Mean age = 61 (SD = 13.4) range 31 - 88	10-item instrument (subscales: relational self-efficacy; instrumental self-efficacy; self-soothing self-efficacy)	5-point Likert scale	Internal consistency per subscale: α = 0.72 relational, α = 0.74 instrumental, α = 0.79 self-soothing subscales. Test–retest reliability (n = 105, 6 months later): r = 0.48–0.69 (p < 0.0001)	Factor structure: 3-factor model confirmed Convergent validity: Instrumental self-efficacy and optimism: r = 0.41, p < 0.001 Self-sooth self-efficacy and social support: r = 0.30, p < 0.001 Relational self-efficacy and coping efficacy = r = 0.32, p < 0.001 Divergent validity: Instrumental self-efficacy and anger: r = -0.35, p < 0.001
Ritter et al. (50), USA	Eight-item caregiver self-efficacy scale (CSES-8)	Intervention caregiver sample (n = 158); Mean age = 65.4 (SD = 10.6), range 23 - 89 Online survey sample (n = 138); Mean age = 78 Supporters of people with cognitive disabilities e.g., stroke, age-related dementia, traumatic brain injury (n of carers of people with dementia not reported)	8-item instrument (caregiving content areas: obtaining respite, controlling negative thoughts, coping with new situations, stress management, self-care, finding resources, and preventing disruptive behaviours)	10-point Likert scale	Internal consistency: In person: α = .89 Online: α = .88 Test-retest in online sample (n=47, 2-3 weeks later): r = 0.75 (p < .001)	Factor structure: PCA identified a single-factor structure. Construct validity: Online study sample with RSCESE (43) subscales: Obtaining respite scale 0.68, p<.001 Responding to disruptive patient behaviour .51, p<.001 Controlling upsetting thoughts .83, p<.001 Construct validity via in person intervention study: Burden: r = -0.66 (p < .001) Depression: r = -0.53 (p < .001) Caregiver strain: r = -0.46 (p < .001) 6-month changes in person sample (n = 117): Burden: r = -0.39 (p < .001) Depression: r = -0.43 (p < .001) Caregiver strain: r = -0.24 (p < .01)
Domain: Resilience and/or Hope						
McGee et al. (51), USA	Resilience Scale 14 (52)	People with early-stage dementia (n=36); Mean age = 74.39 (SD = 10.70), range 56 - 93	14 items	Adapted 3-point Likert scale	Internal consistency: α = 0.81	Convergent validity: Presence of meaning of life: r = 0.48 (p < .01) Optimism: r = 0.38 (p < .05) Life Satisfaction: r = 0.30 (p < .05) Gratitude: r = 0.39 (p < .05) Discriminant validity: Search of meaning life: r = -0.32 (p < .01) Depression: r = -0.54 (p < .01) Anxiety: r = -0.72 (p < .01) Content validity: Only stakeholder engagement with scoring system and administration instructions.
Pione et al. (53), UK	Positive Psychology Outcome Measure – Carer	Family supporters of people living with dementia (n=267); Mean age = 60.51 (SD = 14.37) range 20 - 92	14 item measure (subscales: hope and resilience)	five-point Likert scale	Improved internal consistency for revised PPOM-C: α = 0.948. Hope subscale: α = 0.912; Resilience subscale: α = 0.918 respectively. Test-Retest Reliability: ICC = 0.91 (95% CI = 0.85 – 0.95) over 4 weeks. Hope subscale ICC = 0.891 (95%CI = 0.82 - 0.94). Resilience subscale ICC = 0.874 (95%CI = 0.79 - 0.93).	Convergent validity: Hope subscale: *r* = 0.67 (*p* < .001) Resilience subscale: *r* = 0.58 (*p* < .001) SF-12 Mental Component: *r* = 0.63 (*p* < .001) SF-12 Physical Component: *r* = 0.19 (*p* = .002) Social support: *r* = 0.39 (*p* < .001) Divergent validity: Hospital anxiety and depression: *r* = -0.66 (*p* < .001) Fit Indices (CFA): CFI = 0.904; SRMR = 0.057; RMSEA = 0.114, χ² (340.95, p < 0.001) Content validity: Stakeholder engagement during development (54)
Stoner et al. (55), UK	Positive Psychology Outcome Measure	People living with dementia (n=225); Mean age = 77.1 (SD = 9.4) range 50-99	16 item measure (subscales: hope and resilience)	five-point Likert scale	Internal consistency: Overall PPOM α= .94; hope subscale α=. 88; resilience subscale α =. 92. Test-retest reliability: ICC = .69; after removing two outliers ICC = .88	Convergent validity: QOL (QOL AD; r = .627) and wellbeing (CASP-19; r = .73). Divergent validity: Depression (GDS; r = -.699). EFA indicated a 2-factor model (Hope & Resilience), EFA Eigenvalues: 8.57, 1.14; CFA Fit Indices: Acceptable fit, R² = .55 (Hope), R² = .61 (Resilience) Content validity: Good stakeholder engagement with target user (54)
Hunsaker et al. (56), USA	Hope Herth index	Supporters of people with early cognitive impairment (n=51); Mean age = 74.27 years (SD = 10.15), range 43 – 91 People with cognitive impairment (n=45); Mean age = 70.14 years (SD = 11.49), range 40 - 88	12-item scale (domains measuring: temporality and future; positive readiness and expectanc; and interconnectedness)	Four-point scale	Internal consistency: α = 0.90; Reliability coefficient for Factors 1 and 2 was α = 0.86 and α = 0.83.	Construct validity: Social support: r = .37 (p < .05) Divergent validity: No significant correlation between hope and MMSE, critical illness insight (CIR), or depression in individuals with cognitive impairment. Factor Structure: 2-factor model; EFA Eigenvalues: 5.45, 0.73; Variance Explained: 51.44% Content validity: No stakeholder engagement (57)

The outcome measures identified are organised by domain to facilitate comparison of psychometric properties and ratings. The number of measures identified for each higher-level domain were as follows: hope (3), resilience (2), coping (1), empowerment (3), and self-efficacy in caregivers (6). No outcome measures received a ‘very good’ rating from quality appraisal (see **Table 3** for quality appraisals).

**Table 3:** Quality ratings for the identified outcome measures.

Measure	Content validity	Internal consistency	Criterion validity	Construct validity	Reproducibility agreement	Reproducibility reliability	Responsiveness	Floor/ceiling effect	Interpretability	Total
PPOM-C	2 +	2 +	0 0	2 +	0 0	2 +	0 0	2 +	0 0	10
PPOM	2 +	2 +	0 0	2 +	0 0	2 +	0 0	2 +	1 ?	11
HHI	0 0	2 +	0 0	2 +	0 0	2 +	0 0	0 0	0 0	6
RS14	0 0	1 ?	0 0	1 ?	0 0	0 0	0 0	0 0	0 0	2
EID-Q	2 +	2 +	0 0	2 +	0 0	2 +	0 0	2 +	1 ?	11
CASP-19	0 −	1 ?	0 0	2 +	0 0	2 +	0 0	2 +	0 0	7
DMI	2 +	2 +	0 0	1 ?	0 0	0 0	0 0	0 0	1 ?	6
Brief COPE	0 −	1 ?	0 0	1 ?	0 0	1 ?	1 ?	0 0	1 ?	5
RSCSE	2 +	2 +	0 0	2 +	1 ?	0 0	0 0	0 0	1 ?	8
SES	0 0	0 0	0 0	0 0	0 0	0 0	1 ?	0 0	1 ?	2
SSCQ	0 0	1 ?	0 0	1 ?	0 0	1 ?	0 0	0 0	0 0	3
CGI	1 ?	1 ?	0 0	2 +	0 0	0 0	0 0	0 0	0 0	4
SCQ	0 −	1 ?	0 0	0 −	0 0	0 0	0 0	1 ?	1 ?	3
RIS Eldercare SES	0 0	2 +	0 0	2 +	0 0	0 0	0 0	0 0	0 0	4
CSES-8	1 ?	2 +	0 −	2 +	0 0	1 ?	1 ?	2 +	1 ?	10

Rating: + = positive; 0 = intermediate; − = poor; ? = no information available.

PPOM-C = Positive Psychology Outcome Measure-Carer; PPOM, Positive Psychology Outcome Measure; HHI, Hope Hearth Index; RS14, Resilience Scale 14; EID-Q, Engagement and Independence Questionnaire; CASP-19, Control Autonomy Self-Realisation Pleasure Scale; DMI, Decision-Making Involvement Scale; Brief COPE, Coping Orientation to Problems Experienced; RSCSE, The Revised Scale of Caregiving Self-Efficacy; SES, Self-Efficacy Scale; SSCQ, Short Sense of Competence Questionnaire; CGI, The Caregiver Inventory; SCQ, Sense of Competence Questionnaire; RIS Eldercare SES, RIS Eldercare Self-Efficacy Scale; CSES-8, Eight-Item Caregiver Self-Efficacy Scale.

### Hope in people living with dementia and caregivers

Two outcome measures were identified to assess hope in people living with dementia: the Hope Herth Index (HHI; 56) and the 16-item Positive Psychology Outcome Measure (PPOM; 58), an adapted version of the HHI. The HHI and the 14-item Positive Psychology Outcome Measure-Carer (PPOM-C; 53) were also found to assess hope in family supporters. Both PPOM and PPOM-C contain hope and resilience subscales and have separately established psychometric properties, so they will be described within their respective domains.

The PPOM hope subscale was rated as good (11/18), demonstrating good internal consistency (*α* = 0.88) and moderate reproducibility (ICC = 0.88) and construct validity (quality of life, *r* = 0.60, *p* < 0.001; depression, *r* = −0.68, *p* < 0.001). Factor analysis indicated that PPOM retains a two-factor structure for hope and resilience. The PPOM-C, a 14-item adapted measure for family caregivers, was also rated good (10/18), with factor analysis confirming an improved two-factor structure after removing two items—one item was removed from the hope subscale—to address multicollinearity and improve internal consistency (hope subscale: *α* = 0.91). Additionally, PPOM-C showed good test–retest reliability after 4 weeks (ICC = 0.91) but lacked interpretability. Both measures had high content validity due to substantial involvement from both experts and individuals with lived experience.

The HHI was rated moderate (6/18) due to its good internal consistency (*α* = 0.90) and construct validity, with a positive association with social support (*r* = 0.37, *p* < 0.05). However, there was a lack of data for content validity, interpretability, and floor and ceiling effects when used by people with dementia and family supporters.

### Resilience in people living with dementia

Two measures of resilience for people living with dementia were identified: The Resilience Scale (RS-14), was used with people with dementia in a study providing preliminary psychometric validation of positive psychology measures (51). The PPOM includes a resilience subscale, adapted from established theories and scales such as the Connor Davidson Resilience Scale (59, 60).

The RS-14, a 14-item measure of resilience, showed adequate internal consistency (*α* = 0.81) but was appraised as poor (2/18) due to limited involvement of people with dementia in item selection, small sample size (*n* = 36), and insufficient data on reliability and validity. Factor analysis showed a single dominant resilience factor, confirming unidimensionality and preliminary evidence of convergent (optimism, *r* = 0.38, *p* < 0.05; life satisfaction *r* = 0.30; gratitude *r* = 0.39, *p* < 0.05) and discriminant validity (depression *r* = 0.54, *p* < 0.01; anxiety *r* = −0.72, *p* < 0.01). Internal consistency was found to be good for the 8-item PPOM resilience subscale (*α* = 0.92) when used by people with dementia, demonstrating good convergent validity and discriminant validity (quality of life, *r* = −0.55, *p <* 0.001; depression *r* = −0.70, *p <* 0.001).

The PPOM and PPOM-C received the highest quality ratings (11/18 and 10/18, respectively) for their psychometric properties within the domains of hope and resilience for people with dementia and family supporters.

### Empowerment in people with dementia

Three measures were identified for assessing empowerment: the Engagement and Independence in Dementia Questionnaire (EID-Q; 58), the Decision-Making Involvement Scale (DMI Scale; 41), and the Control, Autonomy, Self-Realisation, Pleasure-19 (CASP-19; 23). All measures were validated for use by people living with dementia and measured various dimensions of empowerment. These include daily decision-making, level of independence, and control of one’s life and environment.

The EID-Q, a 26-item measure with five subscales evaluating activities of daily living, decision-making, activity engagement, support, and reciprocity, was rated good (11/18). It demonstrated good internal consistency (*α* = 0.91) and adequate reproducibility (ICC = 0.79), with high content validity utilising strong stakeholder engagement during its development. The EID-Q significantly correlated with quality of life (*r* = 0.68, *p* < 0.001) and negatively correlated with depression (*r* = −0.74, *p* < 0.001). Factor analysis indicated a two-factor structure capturing both personal and external aspects of empowerment.

The DMI scale measuring different aspects of involvement in decision-making for people with dementia, such as deciding when to get up or financial decision-making, was rated within the moderate quality range (6/18). A separate proxy caregiver measure is available but was excluded from this review. The scale used by people with dementia showed good evidence of internal consistency (*α* = 0.85) and content validity; however, it had weak evidence of construct validity and no evidence of reproducibility.

The CASP-19 contains four subscales assessing control, autonomy, self-realisation, and pleasure and was also rated within the moderate quality range (7/18), with good test–retest reliability (ICC = 0.86). The CASP-19 had poor content validity as it was originally developed with a different population and not adapted before use with people with dementia. The overall internal consistency for CASP-19 was good (*α* = 0.86); however, poor internal consistency was found for key subscales measuring control (*α* = 0.65) and autonomy (*α* = 0.51). There was evidence of adequate convergent validity, being positively correlated with hope and resilience (PPOM; *r* = 0.73), engagement and independence (EID-Q; *r* = 0.75), and quality of life (*r* = 0.71) and negatively correlated with depression (*r* = −0.71).

The EID-Q received the highest rating in the domain of empowerment.

### Coping in caregivers

The Brief COPE (38) was the only identified measure assessing coping strategies in caregivers, evaluating emotion-focussed coping, problem-focussed coping, and dysfunctional coping. It demonstrated moderate psychometric quality (5/18). It also demonstrated moderate construct validity, with emotion-focussed coping significantly associated with problem-focussed coping (*β* = 0.68, *p* < 0.001), whilst dysfunctional coping was significantly predicted by higher caregiver burden (*β* = 0.36, *p* < 0.001). Problem-focussed coping was positively predicted by emotion-focussed coping (*β* = 0.53, *p* < 0.001), dysfunctional coping (*β* = 0.25, *p* = 0.006), and social support (*β* = 0.10, *p* = 0.25). Test–retest reliability was also moderate with total coping scores at time 1 correlating with time 2 and time 3 (*r* = 0.67, 0.54; *p* < 0.001), improving when caregiver burden remained stable (*r* = 0.72, 0.57; *p* < 0.001). However, content validity was poor, lacking involvement from family supporters. Its internal consistency for the three subscales (*α* = 0.72–0.84) was questionable due to a lack of factor analysis. No information was provided regarding floor/ceiling effects and there was minimal important change for measuring clinical significance.

Whilst the Brief COPE was the only coping measure identified, it has poor validity and reliability when used to assess coping in supporters of people with dementia.

### Self-efficacy in family supporters

Six self-efficacy eligible measures were identified: the Revised Scale of Caregiving Self-Efficacy (RSCSE; 43), the Self-Efficacy Scale (SES; 45), the Sense of Competence Questionnaire (SSQ; 48), the Short Sense of Competence Questionnaire (SSCQ; 46), the RIS Eldercare Self-Efficacy Scale (RIS Eldercare; 49), and the Caregiver Self-Efficacy Scale (CSES-8; 50).

### General dementia caregiving self-efficacy measures

The RSCSE (43), rated moderate (8/18), was developed with family supporters of people with dementia to assess caregivers’ confidence in obtaining respite, managing disruptive behaviours, and controlling upsetting thoughts. Factor analysis confirmed a three-factor model fit. It demonstrated good internal consistency (*α* = 0.82–0.85) and test–retest reliability (*r* = 0.70–0.76). Construct validity was adequate, with strong negative correlations with depression (*r* = −0.38) and anger (*r* = −0.45) and a positive correlation with perceived social support (*r* = 0.47).

The CSES-8 (50) was the only self-efficacy measure rated as good (10/18), with subscales evaluating self-efficacy areas such as self-care, obtaining respite, managing stress, and coping with new situations. Adequate internal consistency was demonstrated (*α* = 0.88–0.89). The CSES-8 demonstrated significant negative correlations with caregiver burden (*r* = −0.66, *p* < 0.001), depression (*r* = −0.53, *p* < 0.001), and caregiver strain (*r* = −0.46, *p* < 0.001). Over 6 months, CSES-8 scores remained associated with reductions in burden (*r* = −0.39, *p* < 0.001), depression (*r* = −0.43, *p* < 0.001), and strain (*r* = −0.24, *p* < 0.01). However, content validity was poor as the sample included caregivers of people with dementia along with other various cognitive disabilities such as stroke, with no expert involvement during scale development, limiting its specificity to dementia caregiving. Although the CSES-8 demonstrated criterion validity, it did not correlate strongly with the gold standard RSCSE measure for all subscales (obtaining respite scale 0.68, *p* < 0.001; responding to disruptive patient behaviour 0.51, *p* < 0.001; controlling upsetting thoughts 0.83, *p* < 0.001). Additionally, test–retest reliability was methodologically inadequate with no data found on ICC (*r* = 0.75, *p* < 0.001).

The RIS Eldercare (49), rated poor (4/18), consists of 10 items across three subscales: relational, instrumental, and self-soothing self-efficacy. It demonstrated moderate internal consistency (*α* = 0.72–0.79) and test–retest reliability (*r* = 0.48–0.69, *p* < 0.0001). Construct validity was supported by moderate correlations with optimism (*r* = 0.41, *p* < 0.001) and anger expression (*r* = −0.35, *p* < 0.001).

The SSCQ (46) received a poor quality rating (3/18). There was a good correlation between scores on the SCQ and SSCQ (*r* = 0.88), with good internal consistency (*α* = 0.76). The SSCQ lacked interpretability, responsiveness, floor/ceiling effects, and content validity due to no involvement of informal caregivers during development.

The SES (45), rated 2/18, was developed to assess self-efficacy in a 5-week educational intervention study for family supporters of people with dementia (*n* = 45). The scale demonstrated high internal consistency (*α* = 0.90) but was limited by a lack of factor analysis and inadequate sample size. Although convergent validity data were not available, intervention effectiveness was supported by a significant increase in mean caregiver self-efficacy scores from pre- (*M* = 3.0, SD = 0.7) to post-intervention (*M* = 3.3, SD = 0.7), *t*(43) = −4.619, *p* < 0.0005, with a moderate effect size (*d* = 0.4), demonstrating some responsiveness. However, interpretability remains limited due to a lack of subgroup comparisons and minimum important change (MIC) definitions.

The SSQ (48), evaluated in a sample of caregivers of people with dementia symptoms, was rated 3/18. Though internal consistency was adequate (*α* = 0.83–0.85), the sample size utilised was inadequate for factor analysis (*n* = 93). Construct validity was poor with most *a-priori* hypotheses rejected, and there was presence of ceiling effects.

### Summary of caregiver self-efficacy measures

The CSES-8 demonstrated the highest psychometric quality (good quality; 10/18), with the majority of self-efficacy measures with moderate quality range, and the SES scored the lowest (2/18). The CSES-8 had high reliability and predictive validity over time but has potential limitations in specificity to dementia caregiving due to the mixed caregiver sample and weak criterion validity against the RSCSE. The RSCSE was the only self-efficacy measure with adequate content validity during item selection, and all the other measures either had limited or no involvement by the family supporters or experts. Internal consistency was adequate in all studies, with the RIS Eldercare having the lowest, and the SES having the highest. The SES had no factor analysis performed, so it had questionable internal consistency. Test–retest reliability was performed by only the RIS Eldercare, CSES-8, and RSCSE, but was poorly demonstrated across all measures due to issues regarding the methodology used in studies. Nearly all studies (excluding the SES and SSCQ) had good construct validity, demonstrating significant correlations with constructs like social support, burden, depression, optimism, sense of competence, anxiety, and stress. Data on instrument responsiveness were lacking in all but one study, and all studies failed to provide data for agreement and the value for minimal important change for interpretability. Floor/ceiling effects were not reported for four measures, except for the CSES-8, where information was adequate, and the SSQ, which showed poor effects.

## Discussion

### Key findings

This scoping review highlights the significant gaps in the literature regarding validated outcome measures for evaluating RC dementia courses. Whilst several measures, including the PPOM, PPOM-C, EID-Q (for people with dementia), and CSES-8 (for caregivers) showed promise, these do not address the range of concepts underlying the RC courses in the context of dementia. Our findings highlight the lack of suitable, psychometrically rigorous measures for assessing the impact of these courses for both people with dementia and their family supporters. The review also emphasises the importance of capturing personal recovery through domains such as hope, self-efficacy, resilience, coping, and empowerment, which emerged as key themes from stakeholder discussions. Stakeholders emphasised that the process of regaining control of their lives and maintaining independence and their identities post-diagnosis is an essential recovery outcome for people with dementia. Additionally, self-efficacy and coping were identified as an important domain for supporters of people with dementia, where these strengths were seen as helping to maintain confidence in their caregiving role and support their loved ones appropriately.

Despite the promising findings, none of the reviewed measures received a ‘very good’ rating according to Terwee’s psychometric criteria, with most measures scoring in the poor to moderate range. The only ‘good quality’ rated measures were the PPOM, PPOM-C, EIQ-Q, and CSES-8, which can therefore be tentatively suggested for further psychometric improvement, adaptation, and use (or part-use) to align with endorsements of domains such as hope and empowerment. This further reinforces the need for the development of a high-quality outcome measure for RC dementia courses.

### Strengths

The general mental health RC literature notes challenges in the operational definition of RCs, idiosyncratic practice across organisations delivering RCs, and associated challenges in evaluating success (61). Nonetheless, a strength of this study is our preliminary work to scope strengths-based psychometrically sound instruments for the evaluation of RCs in the UK dementia context. Whilst other similar reviews have addressed the use of evaluating dementia interventions using positive psychology measures (17, 21, 22, 32), this scoping review is the first to explicitly link the positive psychology overlap with the CHIME framework and notions of personal recovery to identify measures for use with post-diagnostic RC dementia courses. Additionally, this was achieved through collaboration with experts and individuals with lived experience. However, the scoping review only offers preliminary answers to the issue of systematically evaluating dementia courses as more development and validation work is required. For example, the content validity for CSES-8 (50) for dementia-family supporters was poor, so it may be unsuitable for the RC dementia course setting. Additionally, although personal recovery and positive psychology tend to focus on personal strengths (as noted also by Stansfield et al., 2017 who examined caregiver instruments), we found that some measures were worded negatively. This is demonstrated by item 13 in the RSCSE (43), “How confident are you that you can control thinking about what a good life you had before :::’s illness and how much you’ve lost?”.

The review also emphasises the importance of stakeholder engagement with people with lived experience in defining meaningful recovery outcomes. People with dementia in the DiSCOVERY Partners in Research stakeholder group identified domains that generic wellbeing measures often overlook, such as empowerment, resilience, and hope. This aligns with the findings from studies using CASP-19 (23), which integrate constructs of control, autonomy, self-realisation, and pleasure into a comprehensive assessment tool of wellbeing; however, this may lack content validity due to the development of the measure with a sample of older people without dementia. A literature review of mental health RCs found very little quantitative evaluation of courses and no outcomes pertaining to empowerment (62). If evaluations are to focus on the important notion of post-diagnosis RC empowerment, we note that the EID-Q (58) as an instrument of ‘good’ quality (albeit requiring more validation work) may have scope for use with people with dementia in RC dementia courses.

Furthermore, stakeholder discussions on hope, self-efficacy, and resilience mirror existing literature within positive psychology in dementia, emphasising psychological and emotional domains (17, 32). This suggests that recovery-oriented frameworks could take precedence in dementia research, moving beyond symptom reduction to focus on holistic outcomes that promote thriving and flourishing despite a dementia diagnosis. Such scales exist in positive psychology, but many have not been used in dementia intervention studies.

Of interest is the notion of stigma in the context of RCs, where this is seen as important in overcoming the psychological and social aspects of a diagnosis (1, 3). In our study, the issue of stigma was noted by stakeholders but did not make the high-level domains for RC dementia outcome measurement. A review by Mast et al. (32) notes one stigma impact instrument with relatively good internal consistency and correlations with high levels of depression and low self-esteem in people with dementia. In terms of measurement for RC dementia courses, stigma may be a mechanism by which people may recover from the shock of their diagnosis (63) and may therefore be relevant as a process rather than an outcome measure.

### Limitations

Previous research has suggested clinical staff attending or co-producing RC courses has been beneficial in shaping their clinical practice and gaining positive attitudes towards recovery-oriented practices (16). This was not addressed in our study which was focussed on the impact for people with dementia or family supporters, since studies referencing co-design/co-production have few co-created evaluations (26). Professional knowledge and related involvement will remain important since many appeared unaware of RCs in the dementia context (10) and links between clinicians who diagnose dementia and an empowerment group for diagnosed people appeared weak (64). Work to capture staff outcomes and the impact of RC dementia courses within organisations and associated practice is indeed warranted.

Limited resources for this review resulted in us not being able to meet one of our objectives, that is, to share the final set of measures identified and fully reflect on the specific content of instruments with DiSCOVERY stakeholders who have lived and learned experience. Therefore, definitive recommendations for use of outcome measures on dementia courses could not be met. Moreover, no one measure received the highest (i.e., very good) rating possible, with only four measures receiving a ‘good’ rating of 10/18 or higher. The measures need further psychometric work, conducted with people with lived experience as co-researchers to ensure content is relevant. Additionally, stakeholders highlighted other important domains for dementia courses to address, including social support, knowledge of dementia, public stigma, and negative attitudes, but relevant outcome measures were not included so as to focus our review on key high-level domains associated with the CHIME framework. Finally, no measure can be considered with regard to family supporter coping as no good quality measures were located.

Another key limitation of this review is the lack of responsiveness data for the identified outcome measures as we did not include intervention studies, unless it included a development or validation process. Responsiveness assesses the ability of a measure to detect meaningful changes over time which is crucial for assessing the long-term effectiveness of RC dementia courses. RC courses are designed to promote recovery and self-management in people with dementia, and responsive measures are necessary to capture how these courses impact attendees over time. Unfortunately, many of the reviewed measures lacked these data, potentially limiting their ability to assess meaningful changes of RC dementia courses. The issue of responsiveness remains a challenge for psychosocial intervention dementia research. For example, in a self-management study, the authors note a small but significant effect of their intervention on an instrument purporting to measure ‘flourishing’ in people with dementia (65). Whilst this could be seen as showing some sensitivity to the intervention, responsiveness (described as longitudinal validity) could not be assumed since Terwee et al. (37) note that detection of an apparent intervention effect on its own does not constitute evidence of responsiveness of a psychometric instrument.

Time limitation did not allow for searching of grey literature. This may have therefore overlooked recent developments in co-produced outcome measures. Many of the measures reviewed were over 10 years old, and it is possible that newer, more relevant tools have emerged since the completion of this review.

The majority of the measures reviewed were developed for broader populations, rather than for attendees of Recovery College courses, suggesting content validity may be lacking. This gap suggests that existing measures may not be fully relevant or sensitive to the specific experiences of people with dementia and their family supporters in an RC context. Therefore, there is a clear need for an adapted measure that accurately reflects domains pertinent to personal recovery in dementia. Three of the four ‘good quality’ outcome measures tentatively suggested have not been translated into alternative languages or validated for use in non-English-speaking countries; therefore, they may lack transcultural relevance. Only the CSES-8 has been translated to Spanish and utilised in a study of Spanish speakers in Latin America (66). There may be very good quality measures of personal recovery developed in other countries and languages which have not been considered in the review due to the exclusion of measures with no English-language versions.

### Implications for practice

Whilst no measure reviewed in this study is currently validated for use in Recovery College dementia courses immediately, some measures, including the PPOM (58), PPOM-C (53), CSES-8 (50), and EID-Q (58), hold promise for further adaptation and validation. These tools could serve as starting points for the development of outcome measures that are more suited to the context of RC dementia courses. However, further psychometric testing is needed to ensure that these measures are sensitive to the changes in personal recovery that occur as a result of attending or co-facilitating dementia courses.

### Future research

Future research should focus on developing recovery-oriented outcome measures that are more in line with the concepts of RC dementia courses. These measures must be sensitive to the personal recovery process pertinent to dementia and the long-term effects of attending these courses for people with dementia and their supporters. Additionally, research should prioritise the development of tools that can capture responsiveness and meaningful changes experienced by people with dementia and their supporters.

There is also a clear need to explore the applicability and feasibility of existing measures, such as the PPOM (58), EID-Q (58), and CSES-8 (50), in the context of RC courses. These measures could potentially be adapted to better reflect the specific outcomes of interest in the context of personal recovery in dementia in collaboration with people with lived experience and their expertise.

## Conclusion

This scoping review highlights the lack of validated outcome measures for evaluating Recovery College dementia courses. Whilst several measures assessing personal recovery and positive psychology domains, such as the PPOM, PPOM-C, EID-Q, and CSES-8 show potential, further psychometric testing and adaptation are required before these tools can be recommended for use in evaluating Recovery College dementia courses. The findings emphasise the need for the development of personal recovery-oriented measures that specifically capture the experiences of people with dementia and their family supporters. Future research should focus on adapting and validating these measures for use on dementia courses to effectively evaluate the impact for attendees.
