# Supplementary material for: A scoping review of outcome measures for people living with dementia and family supporters to evaluate Recovery College dementia courses
Source: Front Psychiatry. 2025 May 6;16:1591772. doi: 10.3389/fpsyt.2025.1591772 (PMC12089082; doi:10.3389/fpsyt.2025.1591772)
Supplement: Supplementary file 1 [file Table1.docx]

**Supplementary File A.** Search strategy for family supporters on APA PsychoInfo

| # | Search Terms | Results |
| --- | --- | --- |
| 1 | dement* OR Alzheimer* [title, subject heading] | 121,444 |
| 2 | famil* OR "family supporter" OR "friend supporter" OR caregiv* OR spouse husband OR wife OR son OR daughter NOT staff [title/ subject heading] | 468,965 |
| 3 | outcome OR measur* OR evaluation OR assessment* OR questionnaire* OR patient-report* OR tool* OR index OR self-report OR scale OR inventor* OR instrument [title and abstract] | 1,813,773 |
| 4 | validation OR develop* OR psychometric [title and abstract] | 955,621 |
| 5 | connect* OR belong* OR social support OR peer OR optimis* OR positive psychol* OR flourish* OR strength* OR resilien* OR hope* OR "education" OR knowledge OR identity OR control OR self-efficacy OR empower* OR stigma OR personhood OR person-cent* OR confiden* OR cop* OR "positive affect" OR purpose [title and abstract] | 1,808,296 |
| 6 | S1 AND S2 AND S3 AND S4 AND S5 | 1439 |
