# Supplementary material for: A scoping review of outcome measures for people living with dementia and family supporters to evaluate Recovery College dementia courses
Source: Front Psychiatry. 2025 May 6;16:1591772. doi: 10.3389/fpsyt.2025.1591772 (PMC12089082; doi:10.3389/fpsyt.2025.1591772)
Supplement: Supplementary file 2 [file Table2.docx]

**Supplementary File B** Search strategy for people with dementia on APA PsychoInfo

| # | Search Terms | Results |
| --- | --- | --- |
| 1 | dement* OR Alzheimer* [title, subject heading] | 129,794 |
| 2 | outcome OR measur* OR evaluation OR assessment* OR questionnaire* OR patient-report* OR tool* OR index OR self-report OR scale OR inventor* OR instrument [title and abstract] | 2225633 |
| 3 | validation OR develop* OR psychometric [title and abstract] | 1198715 |
| 4 | "person-cent*" OR stigma OR self-stigma OR motivat* OR belong* OR flourish* OR ("positive psychological" OR "positive psychologists" OR "positive psychology") OR optimis* OR connect* OR ("social engagement" OR "social engagements") OR "social relationship" OR recover* OR accept* OR agency OR control OR empower* OR self-esteem OR meaning OR purpose OR identity OR strength* OR resilien* OR self-efficacy OR hope* OR education OR knowledge OR peer OR autonomy OR "positive affect" OR self-agency OR self-acceptance OR self [title and abstract] | 2555646 |
| 5 | S1 AND S2 AND S3 AND S4 | 9071 |
