# Supplementary material for: A scoping review of outcome measures for people living with dementia and family supporters to evaluate Recovery College dementia courses
Source: Front Psychiatry. 2025 May 6;16:1591772. doi: 10.3389/fpsyt.2025.1591772 (PMC12089082; doi:10.3389/fpsyt.2025.1591772)
Supplement: Supplementary file 3 [file Table3.docx]

**Supplementary File C** Stakeholder discussions outcome

| **Domain** | **Stakeholder(s)** | **Target User** | **Order of Importance for Family Supporter Measures (based on discussions)** | **Order of Importance for People with Dementia Measures (based on discussions)** |
| --- | --- | --- | --- | --- |
| **Hope** | Staff A, Staff B, Staff C, Partner 1, Partner 2, Partner 3 | Family Supporters/Person with Dementia | 1 | 1 |
| **Cope** | Staff B, Staff C, Partner 3 | Family Supporters/Person with Dementia | 3 | 4 |
| **Self-efficacy/ Competence** | Staff E, Staff C | Family Supporters | 2 | N/A |
| **Empowerment/ Independence/ Autonomy** | Staff B, Staff D, Partner 1, Partner 3, Partner 2, Staff A | Person with Dementia | N/A | 2 |
| **Resilience** | Staff B, Partner 1, Partner 2 | Person with Dementia | N/A | 3 |
| **Stigma/ Attitude** | Staff B, Partner 1, Partner 3 | Family Supporters | 4 (Emphasis on public stigma) | N/A |
| **Social Support** | Staff A, Staff B, Staff C, Partner 3, Partner 1 | Family Supporters/Person with Dementia | 6 | 5 |
| **Knowledge of Dementia** | Staff B, Staff C, Partner 3, Partner 1 (for family supporters), Partner 2 | Family Supporters/Person with Dementia | 5 | 6 |
| **Wellbeing** | X | X | X | X |

**Note:** Rank order: 1 = Most Important, 6 = Least Important. **NB:** The discussion with PiR (Partners in Research) group emphasised that Wellbeing/Quality of life measures may not be suitable for short dementia courses, as they are too broad. The above domains were deemed more relevant to contributing to wellbeing in this context.
